# Questionnaire-based scoring system for screening moderate-to-vigorous physical activity in middle-aged Japanese workers

**DOI:** 10.1093/joccuh/uiad011

**Published:** 2023-11-28

**Authors:** Takuji Adachi, Hironobu Ashikawa, Kuya Funaki, Takaaki Kondo, Sumio Yamada

**Affiliations:** Department of Integrated Health Sciences, Nagoya University Graduate School of Medicine, Nagoya 461-8673, Japan; Program in Physical and Occupational Therapy, Nagoya University Graduate School of Medicine, Nagoya 461-8673, Japan; Program in Physical and Occupational Therapy, Nagoya University Graduate School of Medicine, Nagoya 461-8673, Japan; Department of Integrated Health Sciences, Nagoya University Graduate School of Medicine, Nagoya 461-8673, Japan; Department of Integrated Health Sciences, Nagoya University Graduate School of Medicine, Nagoya 461-8673, Japan

**Keywords:** physical activity, workers, questionnaire, screening

## Abstract

**Objectives:** Currently available questionnaires have limited ability to measure physical activity (PA) using accelerometers as a gold standard. This study aimed to develop a PA questionnaire for middle-aged Japanese workers and propose a PA scoring system for predicting low moderate-to-vigorous PA (MVPA).

**Methods:** A total of 428 participants (median age 49 years; 75.8% men) participated in a 7-day PA measurement using an accelerometer and a questionnaire. The association between questionnaire responses and low MVPA (<150 min/wk) was assessed by logistic regression analysis. A score was assigned to each response based on the correlation coefficients of the multivariate model. The ability of the sum score to predict low MVPA was assessed using the area under the receiver operating characteristic curve (AUC).

**Results:** Five questionnaire items were used for measuring PA scores (range: 0-50; higher scores indicated a higher probability of low MVPA). The AUC was 0.741 (95% CI, 0.689-0.792), and the sensitivity and specificity at the optimal cut-off value were 66.7% and 68.2%, respectively. This predictive ability was slightly increased by body mass index (AUC 0.745 [95% CI, 0.693-0.796]; sensitivity 69.9%; specificity 66.9%). These predictive values were greater than those of conventional questionnaires used in health checkups in Japan (*P* < .05).

**Conclusions:** This questionnaire-based PA scoring system showed moderate accuracy in predicting low MVPA. It is useful for screening physically inactive workers and promoting PA.

## Teaser

**Key points** What is already known on this topic

- Self-administered questionnaires for assessing physical activity are preferred given their suitability for application in large groups.
- These questionnaires have limited ability to measure physical activity using accelerometers as a gold standard because of recall bias, potentially overestimating physical activity.

What this study adds

- In this study, we developed a questionnaire for screening daily moderate to vigorous physical activity (MVPA) among middle-aged Japanese workers and proposed a questionnaire-based scoring system for predicting physically inactive individuals.
- The accuracy of physical activity scores for predicting low MVPA was higher than that of standard questionnaires administered during health checkups in Japan.

How this study might affect research, practice, or policy

- The questionnaire and scoring system proposed in this study is useful for screening physically inactive individuals and promoting MVPA in the field of public health.

## 1. Introduction

Physical activity (PA) is essential for preventing cardiovascular disease, a major health concern worldwide. PA is associated with atherosclerotic risk factors, including obesity, hypertension, and diabetes, increasing the risk of cardiovascular disease.^1^^,^^2^ Additionally, increased shear stress on the vascular wall during PA improves vascular endothelial function.^3^ Therefore, promoting moderate-to-vigorous PA (MVPA) improves public health. The World Health Organization and cardiovascular prevention guidelines recommend performing moderate-intensity PA for at least 150 min/wk or vigorous-intensity PA for at least 75 min/wk.^1^^,^^2^^,^^4^ To achieve the target PA, assessing PA volume and pattern is necessary for planning individualized PA interventions.

**Table 1:** Physical activity-based questionnaire used in the survey.

**Questions**	**Responses**
Q1. Do you walk or ride a bicycle for more than 10 minutes on a one-way commute?	Yes/no
Q2. If “yes,” how much time do you spend walking or biking?	10-15 min/15-20 min/20-30 min/more than 30 min
Q3. Which type of work do you do?	Office work (sitting most of the working hours)/walking and carrying light objects/walking and carrying heavy objects
Q4. If the answer was “office work,” how often do you walk for 5 minutes during a usual half-day work?	Never/once/twice/three times/four times/more than five times
Q5. In a typical week, how much time do you spend performing housework, sports (walking outside, jogging, or gym workout), gardening, and other activities?	None/less than 60 min/60-90 min/90-150 min/150-300 min/more than 300 min
Q6. How often do you feel out of breath during physical activity?	Rarely/sometimes/often/very often
Q7. How long have you been performing physical activity?	Less than 6 months/6 months to 1 year/1-3 years/more than 3 years

Movement sensors such as accelerometers are widely used in PA research owing to their analytical accuracy. However, using accelerometers is not feasible in large-scale surveys, including cohort studies and group health checkups, due to the associated high cost. Hence, self-administered questionnaires are preferred given their suitability for application in large groups. By contrast, these questionnaires have limited ability to measure PA using accelerometers as a gold standard because of recall bias, potentially overestimating PA.^5^ Therefore, the ease of recalling daily PA is crucial for developing questionnaires for PA assessment.

Daily PA includes walking and other activities of daily living. Questionnaires that classify daily PA into different categories, including commuting time, walking during work, and leisure-time activity, are completed more efficiently, thus reducing recall bias. This questionnaire structure can better identify PAs that require improvement. Additionally, given the difficulty of describing the total time of PA owing to recall bias, screening physically inactive individuals may be more feasible than calculating the estimated time of PA. Therefore, we aimed to develop a PA questionnaire for middle-aged Japanese workers and propose a PA scoring system for predicting an MVPA of <150 min/wk measured using an accelerometer.

## 2. Methods

### 2.1. Study population

The participants were employees of a parent company and independent affiliated companies in Japan. Most companies were involved in the production of precision machinery and industrial products. Individuals who underwent hemodialysis, exhibited ambulatory limitations, or experienced unique circumstances during the PA measurement period (eg, scheduled business trips, paid leave, or vacation) were excluded. The participants were recruited by sending emails and posting announcements on online bulletin boards. All participants provided written informed consent.

### 2.2. PA-based questionnaire

The questionnaire was developed through a literature review and expert panel discussion. Two physical therapists and 1 physician joined the expert panel. These professionals had extensive clinical experience and expertise in conducting clinical research on cardiovascular prevention and public health. Several questionnaires were analyzed during the literature review.^6-11^ Our questionnaire met the following requirements: (1) ability to measure MVPA, (2) ease of recalling and answering, (3) a small number of items and scales, and (4) applicability for tailored home-based PAs.

Based on these requirements, questions related to commuting time, walking during work, and leisure-time activity were formulated. The contribution of walking to work to daily MVPA levels was assessed.^12^ The questionnaire items and answers were further refined by conducting a preliminary survey. The participants were asked to answer the questions online and describe items that were difficult to understand or answer. Based on these results, we clarified the questions and modified the answers to reduce the ceiling and floor effects. The final version of the questionnaire is presented in Table 1.

The following 3 questions were asked: (1) “Do you perform moderate-intensity exercises for over 30 minutes per session, twice weekly, for over a year?” (2) “In your daily life, do you walk or engage in any PA for more than 1 hour a day?” and (3) “Is your walking speed faster than the speed of people your age and sex?” These items were included in the standard questionnaire used for health checkups recommended by the Japanese Ministry of Health, Labour, and Welfare. We assessed these conventional items to compare the predictive accuracy for low MVPA using the PA scores developed in this study. Each item was answered with a yes (1 point) or no (0 point), and the sum score was calculated for each participant (range: 0-3).^13^

### 2.3. Measurement of daily PA

The daily PA of each participant was measured for 7 consecutive days using an electrical accelerometer (Kenz Lifecorder GS, Suzuken, Nagoya, Japan; 45 g; width 72.0 mm; height 42.0 mm; thickness 29.1 mm). The device samples uniaxial (vertical) accelerations at a rate of 32 Hz, and a maximum pulse over 4 seconds is recorded as the acceleration value. PA intensity was categorized into 11 levels (0, 0.5, and 1-9) based on the acceleration pattern. An acceleration intensity of >4 was considered MVPA (activity at an intensity of >3 metabolic equivalents).^14^ This device has good reliability and validity.^15^^,^^16^ Kenz Lifecorder has higher validity than some other wearable devices but tends to underestimate PA especially during free-living conditions or nonlocomotive activities.^17^^,^^18^

All measurements were performed in spring to avoid seasonal variations in PA. The duration of MVPA and step count for each participant were calculated. The participants were instructed to wear the accelerometer on their waist for 24 h/d for 1 week, except during bathing and sleeping, and to perform daily activities as usual. The participants were blinded to their daily step count and MVPA levels within 7 days to avoid measurement bias. Data monitored for ≥10 h/d were included in the analysis.^19^ Wear time was calculated by subtracting non-wear time (at least 20 consecutive minutes of zero activity intensity) from 24 hours.^19^ The MVPA and step counts were calculated within ≥5 valid days.^20^ Data obtained within ≤4 valid days were regarded as missing data.

### 2.4. Participant characteristics

Japanese law requires business operators to conduct health checkups of their employees annually. Data on age, sex, body mass index (BMI), blood pressure levels, lipid profile levels, fasting blood glucose levels, and hemoglobin A1c levels were collected during checkups. Data on prescribed drugs—antihypertensive, lipid-lowering, and antidiabetic drugs—were also obtained from medical records. In Japan, medical receipts describing medical treatments administered to patients and corresponding fees are collected monthly by health insurance unions. The study participants had standard insurance coverage and did not undergo additional health checkups.

**Figure 1:**
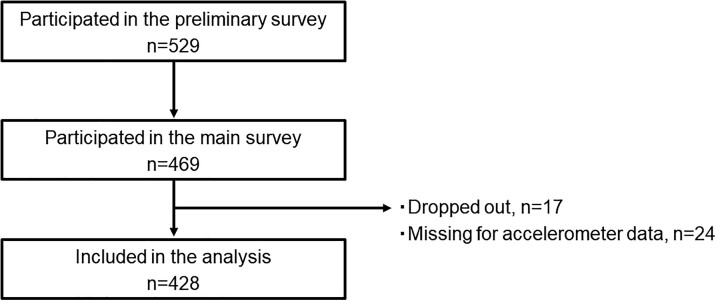
Flowchart of the study selection process.

**Table 2:** Characteristics of the study participants.

**Characteristic**	**Value**
Age, y	49 (43-54)
Men, %	75.8
Body mass index, kg/m^2^	22.5 (20.7-24.7)
<25, %	76.0
25 to <30, %	18.9
≥30, %	5.1
Systolic blood pressure, mmHg	118 (110-126)
Diastolic blood pressure, mmHg	74 (67-81)
Triglycerides, mg/dL	76 (55-115)
HDL-C, mg/dL	60 (51-71)
LDL-C, mg/dL	121 (98-139)
Fasting blood glucose, mg/dL	89 (84-95)
HbA1c, %	5.4 (5.2-5.5)
Antihypertensive drugs, %	11.7
Lipid-lowering drugs, %	7.9
Antidiabetic drugs, %	2.3
Type of work, %	
Office work (sitting most of the working hours)	90.2
Walking and carrying light objects	2.6
Walking and carrying heavy objects	7.2
MVPA, min/d	30.0 (19.8-43.6)
MVPA, min/wk	210.2 (138.7-305.2)
<75, %	7.5
75-149, %	21.2
150-299, %	45.1
≥300, %	26.2
Step counts, steps/d	8129 (6270-10 497)

Continuous variables are expressed as the median (interquartile range).

Abbreviations: HbA1c, glycated hemoglobin; HDL-C, high-density lipoprotein cholesterol; LDL-C, low-density lipoprotein cholesterol; MVPA, moderate-to-vigorous physical activity.

**Table 3:** Results of the logistic regression analysis, including questionnaire responses.

**Variables included in the model** ^**a**^	**Correlation coefficient**	**95% CI**	***P* value**	**Assigned score** ^**b**^
**Commutes neither by walking nor by bicycle**	1.25	(0.66 to 1.84)	<.001	13
**Frequency of walking at work**				
**Carrying objects**	(Ref)			0
**Office work, walk 3 or 4 times**	0.43	(−0.47 to 1.34)	.347	4
**Office work, walk twice**	0.47	(−0.44 to 1.37)	.310	5
**Office work, walk once or never**	0.87	(0.01 to 1.73)	.047	9
**Duration of leisure-time physical activity (min/wk)**				
**≥300**	(Ref)			0
**150–300**	1.43	(0.44 to 2.41)	.005	14
**90–150**	1.20	(0.18 to 2.23)	.022	14
**60–90**	1.76	(0.77 to 2.76)	.001	18
**<60**	1.82	(0.83 to 2.82)	<.001	18
**Not engaged in leisure-time physical activity**	2.78	(1.69 to 3.87)	<.001	28
**Frequency of light breathlessness during leisure-time physical activity**				
**Often or very often**	(Ref)			0
**Sometimes**	0.34	(−0.23 to 0.92)	.242	3
**Rarely**	0.60	(−0.10 to 1.35)	.009	6
**Not engaged in leisure-time physical activity**	Omitted			—

a Dependent variable: moderate-to-vigorous physical activity for less than 150 min/wk.

b The assigned score was the regression coefficient of each variable multiplied by 10.

### 2.5. Statistical analysis

Participants with missing data were excluded from the analysis. Continuous variables were expressed as the means and SDs for normally distributed variables and as the medians with interquartile ranges for nonnormally distributed data. Categorical data were expressed as numbers and percentages.

Low MVPA was defined as an MVPA of <150 min/wk that did not achieve the PA levels recommended by the World Health Organization and cardiovascular prevention guidelines.^1^^,^^2^^,^^4^ The association between questionnaire responses and low MVPA was evaluated using univariate logistic regression. Based on the distribution of responses and the results of the univariate logistic regression analysis, we selected the questions to be included in the multivariate analysis and categorized the answers to improve the accuracy of low MVPA prediction.

Multivariate logistic regression analysis was performed using an MVPA of <150 min/wk as a dependent variable and questionnaire responses as independent variables. According to the correlation coefficients of the logistic regression model, a score was assigned to each variable to reflect the weight of each response for low MVPA, and the sum of the scores was considered the PA score (PA score 1). The assigned score was the regression coefficient of each variable multiplied by 10.^21^ In addition to this basic model, logistic regression analysis was performed, adding age, sex, and BMI to calculate another PA score considering these variables (PA score 2). This additional score was calculated because these variables could be collected during health checkups and considered a proxy for MVPA.^22^ The multivariate logistic regression models were internally validated using the standard bootstrap resampling method recommended for predictive models.^23^ Moreover, the small sample size used in the data-splitting method supported bootstrap resampling for internal validation. The Spearman’s rank correlation coefficients of PA scores were calculated, and an accelerometer was used for measuring the MVPA.

The predictive accuracy of PA scores for an MVPA of <150 min/wk was assessed by conducting a receiver operating characteristic (ROC) curve analysis. The area under the ROC curve (AUC) was calculated and compared using the method employed in DeLong et al’s study.^24^ The optimal cut-off PA scores were identified based on the Youden index.

All statistical analyses were performed using Stata/SE software version 15.1 (StataCorp LP, College Station, TX, USA). A *P* value <.05 was considered significant.

### 2.6. Ethical statement

This study was conducted in accordance with the principles of the Declaration of Helsinki and approved by the Research Ethics Committee of the School of Health Sciences, Nagoya University (approval number: 21–506). All participants provided written informed consent.

## 3. Results

A total of 469 workers from 16 of 47 prefectures in Japan participated in this study. Of them, 428 workers were included in the analysis after excluding those who were lost to follow-up and with missing data (Figure 1). The median age was 49 years (interquartile range 43-54), and 75.8% were men. The characteristics of the study participants are presented in Table 2. The median MVPA and step count were 30.0 min/d (interquartile range 19.8-43.6) and 8129 steps/d (interquartile range 6270-10 497), respectively. Approximately 90% of the participants were involved in office work (sitting most of the working hours). The prevalence of engaging in MVPA of <150 min/wk was 28.7%.

After performing a univariate logistic regression analysis, we selected the questions to be included in the multivariate analysis for predicting an MVPA of <150 min/wk. The multivariate model that contained questionnaire responses is shown in Table 3. The model did not include the time of PA during commuting owing to the lack of association between self-reported and actual MVPA (Figure SS1). The logistic regression analysis is shown in Table 3. A score corresponding to the adjusted coefficient for each response was assigned; the total score (PA score 1) ranged from 0 to 50, and higher scores indicated a higher probability of engaging in an MVPA of <150 min/wk. However, the multivariate analysis results did not considerably change after excluding participants who used a bicycle for their daily commute to work (*n* = 23). Although the probability of low MVPA was lower in participants with 90-150 min/wk of leisure-time activity than in those with 150-300 min/wk in the multivariate analysis, the same score was assigned to the 2 responses based on the distribution of PA (Figure SS2).

The multivariate model is shown in Table 4. Age and gender were excluded from the model owing to the lack of association with low MVPA in the univariate logistic regression analysis. A BMI of ≥30 kg/m^2^ was associated with low MVPA, whereas other BMI categories were not and were excluded from the model. The total score range derived from this model (PA score 2) was 0-59. The Spearman’s rank correlation coefficients of PA scores 1 and 2 with actual MVPA were −0.438 (*P* < .001) and −0.446 (*P* < .001), respectively.

**Figure 2:**
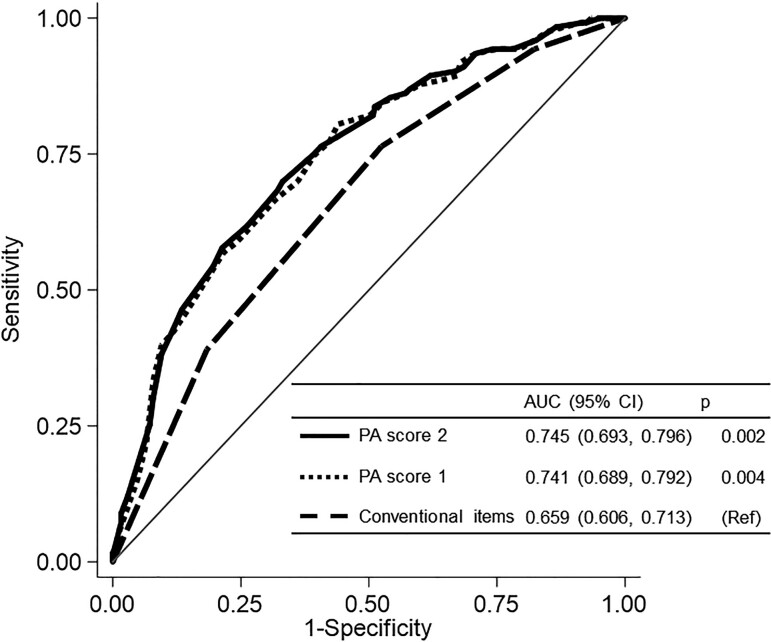
Results of the receiver operating characteristic curve analysis. Dependent variable: moderate-to-vigorous physical activity for less than 150 min/wk. PA score 1 was calculated based on the questionnaire responses. PA score 2 was calculated based on the questionnaire responses and body mass index data. AUC, area under the receiver operating characteristic curve; PA, physical activity.

The ROC curves for predicting an MVPA of <150 min/wk are presented in Figure 2. The AUC of PA score 1 was 0.741 (95% CI, 0.689-0.792), and the sensitivity and specificity of the optimal cut-off value (36 points) were 66.7% and 68.2%, respectively (Table SS1). The AUC of PA score 2 was 0.745 (95% CI, 0.693-0.796), and the sensitivity and specificity of the optimal cut-off value (36 points) were 69.9% and 66.9%, respectively. The AUC of conventional items was 0.659 (95% CI, 0.606-0.713), and the sensitivity and specificity of the optimal cut-off value (2 points) were 76.4% and 47.5%, respectively. The AUC of PA scores 1 and 2 was significantly higher than that of the conventional items (*P* = .004 and *P* = .002, respectively).

## 4. Discussion

In this study, we developed a questionnaire for screening daily MVPA among middle-aged Japanese workers and proposed a questionnaire-based scoring system for predicting physically inactive individuals (Table SS2). The PA score showed a moderate correlation with accelerometer-based MVPA data and moderate accuracy in predicting low MVPA. The accuracy of PA scores slightly increased after adding BMI. The predictive accuracy of PA scores was higher than that of standard questionnaires administered during health checkups in Japan. Although external validation was not performed, the questionnaire is useful for screening physically inactive individuals.

MVPA is a widely accepted indicator of PA for preventing major noncommunicable diseases. One in 4 adults performs <150 min/wk of MVPA, which is less than the globally recommended level to achieve health benefits.^25^ Although recent guidelines have focused on decreasing sedentary behavior, promoting MVPA is a core component of a healthy lifestyle. Middle age is a critical stage for maintaining physical activity to enhance health and reduce the risk of diseases and disability in older age. Increased PA in middle age delays the onset of disability by as much as 15 years.^26^ A previous study reported that a higher PA level during middle age is associated with better perceived health in older age.^27^ Therefore, questionnaires for assessing MVPA can promote an active lifestyle in middle-aged workers.

**Table 4:** Results of the logistic regression analysis after including participant characteristics.

**Variables included in the model** ^**a**^	**Correlation coefficient**	**95% CI**	***P* value**	**Assigned score** ^**b**^
**Commutes neither by walking nor by bicycle**	1.26	(0.61 to 1.91)	<.001	12
**Frequency of walking at work**				
**Carrying objects**	(Ref)			0
**Office work, walk three or four times**	0.49	(−0.57 to 1.55)	.361	5
**Office work, walk twice**	0.53	(−0.51 to 1.58)	.316	5
**Office work, walk once or never**	0.97	(−0.05 to 2.00)	.051	10
**Time of leisure-time physical activity, min/wk**				
**≥300**	(Ref)			0
**150–300**	1.43	(−0.55 to 3.43)	.157	14
**90–150**	1.20	(−0.81 to 3.20)	.244	14
**60–90**	1.70	(−0.28 to 3.66)	.093	18
**<60**	1.79	(−0.19 to 3.78)	.077	18
**Not engaged in leisure-time physical activity**	2.75	(0.75 to 4.74)	.007	28
**Frequency of light breathlessness during leisure-time physical activity**				
**Often or very often**	(Ref)			0
**Sometimes**	0.33	(−0.27 to 0.093)	.276	3
**Rarely**	0.62	(−0.17 to 1.41)	.120	6
**Not engaged in leisure-time physical activity**	Omitted			—
**Body mass index ≥30 kg/m** ^**2**^	0.85	(−0.17 to 1.90)	.098	9

a Dependent variable: moderate-to-vigorous physical activity for less than 150 min/wk.

b The assigned score was the regression coefficient of each variable multiplied by 10.

The most important feature of the proposed questionnaire is the classification of daily PA into commuting time, walking during work, and leisure-time PA, thus reducing recall bias. Previous meta-analyses have reported a low to moderate correlation between some PA questionnaires and accelerometer-based MVPA data, including the International Physical Activity Questionnaire (31 items) (*r* = 0.27-0.66),^6^ International Physical Activity Questionnaire-Short Form (9 items) (*r* = −0.03 to 0.34),^8^ and Global Physical Activity Questionnaire (16 items) (*r* = 0.10-0.48).^7^ The correlation coefficient between questionnaire-based PA scores and actual MVPA metrics was −0.438, which was in line with the findings of previous studies. Given that the final version of our questionnaire requires respondents to answer 5 multiple-choice questions, we consider this correlation with MVPA to be acceptable. Moreover, the PA pattern of workers can be assessed in 3 domains: commuting, working, and leisure-time PA. This feature helps set action goals to increase PA levels and has been incorporated in PA questionnaires that estimate the total PA volume.

The predictive accuracy of the PA score 1 for low MVPA slightly increased after adding BMI (PA score 2). Previous studies have reported that those with a higher BMI performed less PA than those with a lower BMI.^28^^,^^29^ In this study, we proposed using the model containing BMI owing to the potential contribution of this index to MVPA prediction, although the association with MVPA was not significant in the multivariate analysis. The predictive accuracy of PA scores was higher than that of standard questionnaires used in Japan. This finding demonstrates the potential usefulness of this questionnaire in routine practices such as group health checkups. Future research should assess the relationship of PA scores with health conditions, including the risk of cardiovascular disease.

The generalizability of the study findings should be carefully discussed. Although the prevalence of low MVPA (28.7%) in this study was similar to that reported in previous studies conducted in foreign countries,^25^ the median step count of 8129 in this study was higher than that of the general population with a similar age in Japan (6500-7000 steps/d).^30^ This result was possibly attributed to the inclusion of physically active volunteers, suggesting the risk of selection bias. Additionally, a previous study reported that the step count calculated by Kenz Lifecorder was higher than that measured by a pedometer with a spring-mass system used in the National Health and Nutrition Survey in Japan.^31^ Hence, the difference in devices used for PA measurement may also explain the difference in PA levels between the present study and the nationwide survey in Japan.

This study has some limitations. First, people interested in health were more likely to be enrolled in the study than the general population since participation was voluntary. Although the prevalence of low MVPA corroborated with that reported in previous studies,^25^ the median step count in this study was higher than that of the general population with a similar age in Japan.^30^ Additionally, the prevalence of obesity at baseline in our cohort (25.5% in men, 19.6% in women) was lower than that in the same age group in the Japanese population.^32^ Second, the study participants were recruited from a single company and its affiliated companies. The participants consisted primarily of men, and approximately 90% were involved in office work. Due to the above reasons, a risk of selection bias exists, and the generalizability of the findings of this study should be carefully considered. Third, external validation was not performed. Therefore, the questionnaire and PA scores are still in the preliminary phase, limiting the generalizability of the findings. Fourth, although the participants were blinded to the PA measurements and were instructed to perform the activities of daily living as usual during the study period, wearing of the accelerometer may encourage PA.

In conclusion, we proposed a PA questionnaire to screen middle-aged Japanese workers with low MVPA. The questionnaire-based PA scores showed a moderate correlation with accelerometer-based MVPA data and moderate accuracy in predicting low MVPA. Although external validation was not performed, the proposed questionnaire is useful for screening physically inactive individuals and promoting PA.

## Author contributions

S.Y. and T.A. contributed to the conception, design of the study, and interpretation and analysis of the data. H.A. and K.F. contributed to the acquisition and interpretation of data for the work. T.K. contributed to the analysis and interpretation of the data for this work. T.A. drafted the manuscript. All authors critically revised the manuscript, approved the final manuscript, and agreed to be accountable for all aspects of the work, ensuring its integrity and accuracy.

## Funding

This study was supported by EPSON Health Insurance Association. The funders contributed to the participant recruitment and the online questionnaire development but were not involved in the study design and data analysis. This work was also partly funded by JSPS KAKENHI (grant number: 23 K16769), and the funder was not involved in the study design or analysis.

## Conflicts of interest

The authors declare that they have no conflicts of interest.

## Data availability

The data that support the findings of this study will be shared on reasonable request to the corresponding author. The data are not publicly available due to privacy requirements and ethical restrictions.

## Supplementary Material

Web_Material_uiad011
