# Supplementary material for: Questionnaire-based scoring system for screening moderate-to-vigorous physical activity in middle-aged Japanese workers
Source: J Occup Health. 2023 Nov 28;66(1):uiad011. doi: 10.1093/joccuh/uiad011 (PMC11254300; doi:10.1093/joccuh/uiad011)
Supplement: Web_Material_uiad011 [file web_material_uiad011.zip › Supplementary Table 1_R1.docx]

**Supplementary information**

**Supplementary Table 1.** Sensitivity and specificity for predicting moderate-to-vigorous physical activity for less than 150 min/week according to different cut-off values.

| Cut-off | Sensitivity | Specificity |
| --- | --- | --- |
| PA score 1 |  |  |
| 35 | 69.9% | 63.9% |
| 36 | 66.7% | 68.2% |
| PA score 2 |  |  |
| 36 | 69.9% | 66.9% |
| 37 | 68.3% | 67.9% |
| 38 | 61.8% | 73.8% |
| Conventional items |  |  |
| 2 | 76.4% | 47.5% |
| 3 | 39.0% | 81.6% |
