# Supplementary material for: Questionnaire-based scoring system for screening moderate-to-vigorous physical activity in middle-aged Japanese workers
Source: J Occup Health. 2023 Nov 28;66(1):uiad011. doi: 10.1093/joccuh/uiad011 (PMC11254300; doi:10.1093/joccuh/uiad011)
Supplement: Web_Material_uiad011 [file web_material_uiad011.zip › Supplementary Table 2_R1.docx]

**Supplementary information**

**Supplementary Table 2.** Final version of the physical activity questionnaire.

| Questions | Responses |
| --- | --- |
| Q1. Do you walk or ride a bicycle for more than 10 minutes on a one-way commute? | Yes/no |
| Q2. Which type of work do you do? | Office work (sitting most of the working hours)/ walking and carrying light objects/walking and carrying heavy objects |
| Q3. If the answer was “office work,” how often do you walk for 5 minutes during a usual half-day work? | Never/once/twice/three times/four times/more than five times |
| Q4. In a typical week, how much time do you spend performing housework, sports (walking outside, jogging, or gym workout), gardening, and other activities? | None/less than 60 min/60-90 min /90-150 min/150-300 min/more than 300 min |
| Q5. How often do you feel out of breath during physical activity? | Rarely/sometimes/often/very often |

Japanese version（日本語版）

| 設問 | 回答肢 |
| --- | --- |
| Q1. 片道の通勤で「連続して10分以上」歩いているまたは自転車に乗っていますか？ | はい/いいえ |
| Q2. あなたの仕事中の作業形態を教えてください。 | 多くの時間座っている/多くの時間、歩いたり、軽い物を運んでいる/多くの時間、歩いたり、重いものを運んでいる |
| Q3. 質問3で「多くの時間座っている」と回答された方にお伺いします。平均的な半日の仕事中（3時間程度）に、5分程度の歩行をすることは何回ありますか？ | なし/1回程度/2回程度/3回程度/4回程度/ 5回以上 |
| Q4. 平均的な週において、家事やスポーツ（散歩、事務での運動、ジョギングなどの趣味活動）、庭や畑仕事など、「身体を動かす活動」は1週間に合計してどのくらい行っていますか？ | 行っていない/60分未満/60～90分/90～150分/150～300分/300分以上 |
| Q5. 質問4でお答えいただいた「身体を動かす活動」中に、息が弾むことはどの程度ありますか？ | ほとんどない/少しある/しばしばある/かなりある |
