# Supplementary figures and images for: Questionnaire-based scoring system for screening moderate-to-vigorous physical activity in middle-aged Japanese workers
Source: J Occup Health. 2023 Nov 28;66(1):uiad011. doi: 10.1093/joccuh/uiad011 (PMC11254300; doi:10.1093/joccuh/uiad011)

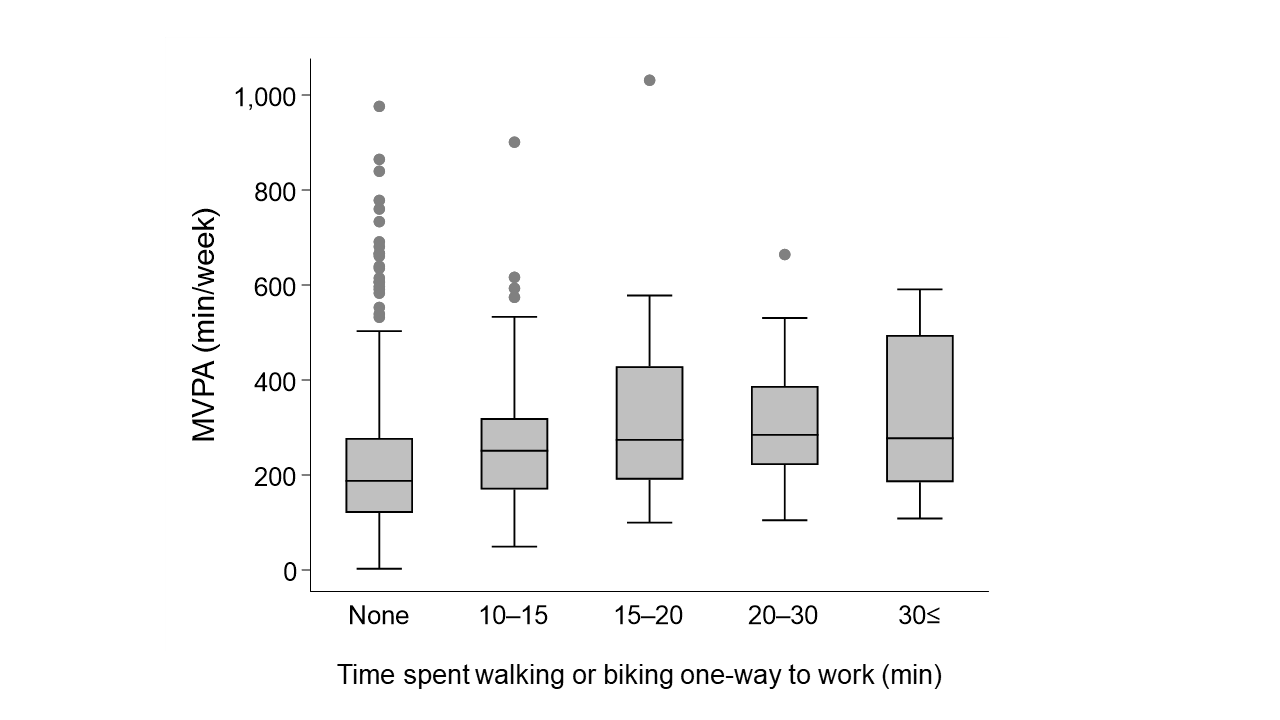

Supplement: Web_Material_uiad011 [file web_material_uiad011.zip › Suppl Figure 1.TIF]

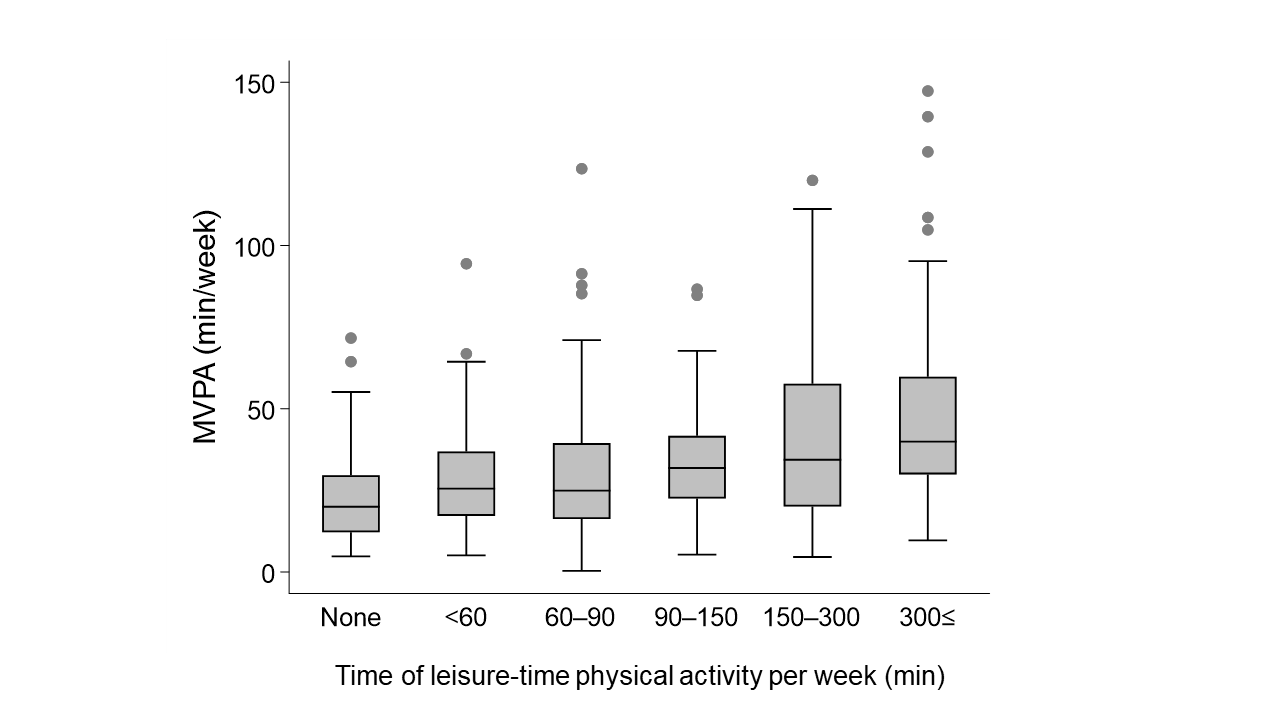

Supplement: Web_Material_uiad011 [file web_material_uiad011.zip › Suppl Figure 2.tif]
